# Early and Late Outcomes after Arterial Switch Operation: A 40-Year Journey in a Single Low Case Volume Center

**DOI:** 10.3390/medicina57090906

**Published:** 2021-08-30

**Authors:** Karolis Jonas, Virginijus Jakutis, Rita Sudikienė, Virgilijus Lebetkevičius, Giedrius Baliulis, Virgilijus Tarutis

**Affiliations:** 1 Center of Cardiothoracic Surgery, Clinic of Cardiovascular Diseases, Institute of Clinical Medicine, Vilnius University Faculty of Medicine, Santariskiu St. 2, LT-08661 Vilnius, Lithuania; rita.sudikiene@santa.lt (R.S.); virgilijus.lebetkevicius@santa.lt (V.L.); virgilijus.tarutis@santa.lt (V.T.); 2 Clinic of Anesthesiology and Intensive Care, Institute of Clinical Medicine, Vilnius University Faculty of Medicine, Santariskiu St. 2, LT-08661 Vilnius, Lithuania; virginijus.jakutis@santa.lt; 3 Department of Cardiac Surgery, University Hospital Southampton NHS Foundation Trust, Trenona Road, Southampton SO16 6YD, UK; giedrius.baliulis@uhs.nhs.uk

**Keywords:** early and late outcomes of arterial switch operation, low case volume congenital heart surgery, sustainability of international knowledge transfer in complex congenital heart surgery

## Abstract

*Background and Objectives*: The results of the arterial switch operation in large congenital heart centers are excellent, and the results in small and medium centers are improving. The objective of this article is to share our experience utilizing the international knowledge transfer program to improve early and late arterial switch operation outcomes in our center. *Materials and Methods*: A retrospective analysis of patients who underwent the arterial switch operation in Vilnius University Santaros Clinics Cardiothoracic Surgery Center between 1977–2020 was performed. *Results*: A total of 127 consecutive arterial switch operations were performed in our center. Surgical mortality during the entire study period was 24.6%. Surgical mortality prior to the program, during the program, and after the program was 88.24%, 41.7%, and 5.81%, respectively (*p* < 0.0001). The surgical mortality of patients operated on during the last 10 years was 4%. The overall survival estimate for the 97 surviving patients was 96.9%, 94.9%, 93.8%, 93.8%, 93.8%, 93.8% at 1, 3, 5, 10, 15, and 20 years, respectively. Risk factors for early mortality included longer aortic cross-clamp time and operation prior to the knowledge transfer program. The only significant risk factor for late reintervention was concomitant aortic arch obstruction treated at the time of the arterial switch. *Conclusions*: The surgical treatment of transposition of the great arteries by means of an arterial switch with good results can be possible in low-to-medium volume congenital heart surgery centers. International knowledge transfer programs between high-expertise high-volume congenital heart centers and low-to-medium volume congenital heart centers may help to shorten the learning curve and improve early and late outcomes after an arterial switch. The risk factors for surgical mortality and intervention-free survival in low-volume surgical centers are similar to those in high-volume centers. Late arterial switch-related complications are similar to those among different-sized congenital heart centers.

## 1. Introduction

The discordant ventriculoarterial connection was first described by Baillie in 1797 and named transposition of the great arteries by Farre in 1814 [1,2]. Currently, the arterial switch operation, as described by Jatene with the Lecompte maneuver, is considered the gold standard in treating transposition of the great arteries [3,4]. The arterial switch operation, when performed in large congenital heart centers, has shown very good results [5,6,7,8,9,10,11,12,13]. However, it is not always possible to refer the patient for treatment in a large center, as illustrated by the international travel halt due to the coronavirus 2019 (COVID-19) pandemic. On the other hand, the increasing number of small and medium congenital heart surgery centers have reported improving arterial switch operation survival results [14,15]. In this article, we analyze our small-volume center results of treating patients with transposition of the great arteries.

## 2. Materials and Methods

A retrospective analysis of patients treated for various forms of transposition of the great arteries from 1977–2020 at Vilnius University Hospital Santaros Clinics Cardiothoracic Surgery Center was conducted. The study was approved by a local ethics committee. Only patients who underwent arterial switch operations were included in this study. In total, 127 patients underwent arterial switch operations during the study period in our center. The cohort was comprised of three groups according to the form of the transposition: patients with transposition of the great arteries and intact ventricular septum (*n* = 82); patients with transposition of the great arteries and ventricular septal defects (*n* = 30); and patients with a Taussig-Bing anomaly (*n* = 15). Medical charts were revised, and patient demographic, pre-operative, operative, and follow-up data were gathered. Early post-operative mortality was analyzed retrospectively, and surviving patients were followed for late morbidity and mortality. Z-scores for all echocardiographic parameters were calculated using regression formulas from the Detroit Data group [16]. A compound endpoint of first intervention and death was chosen as an event for intervention-free survival. A definition of interventions was chosen to include only major surgical or catheter-based cardiothoracic interventions. Delayed chest closure, emergency chest opening during a cardiovascular collapse for open-chest cardiopulmonary resuscitation, insertion or removal of a temporary peritoneal dialysis catheter, and insertion or removal of chest drains were excluded from the definition of interventions.

### 2.1. Statistical Analysis

The distribution of numerical variables was tested by comparing the central tendency of the data, visual analysis of the histogram, and using statistical normality tests (Shapiro-Wilk and Kolmogorov-Smirnov). Numerical variables are presented as median with interquartile range (IQR), or just the range where applicable—as all numerical data were not normally distributed. All categorical variables are presented as counts and percentages. Numerical variables between groups were compared using the Mann-Whitney-Wilcoxon test or Kruskal-Wallis rank-sum test where applicable. Categorical variables between groups were compared by χ^2^ or Fisher’s exact tests where applicable. Survival analysis was performed using the Kaplan–Meier method. A log-rank test was used to compare different survival estimates. Risk factors for surgical mortality and the need for late interventions were determined using a logistic regression model. Factors with a *p*-value < 0.2 in the univariable analysis were included in the subsequent multivariable analysis. The significance of these factors was then tested using backward stepwise regression. A *p*-value < 0.05 was chosen to represent statistical significance. Statistical analysis was performed using R statistical software package version 3.4.4 (R Core Team, R Foundation for Statistical Computing: Vienna, Austria, 2018) [17].

### 2.2. Operative Technique and Myocardial Protection

All patients underwent a standard arterial switch operation with the Lecompte maneuver, where possible. The first two procedures were performed using deep hypothermic cardiac arrest without coronary perfusion while maintaining systemic circulation with cardiopulmonary bypass. The heart was arrested using topical cooling with ice slush. All other procedures prior to the international knowledge transfer program were performed using crystalloid cardioplegia and moderate systemic hypothermia. During the knowledge transfer program, myocardial protection was switched to intermittent cold blood cardioplegia and mild to moderate systemic hypothermia. This technique is used to this day. Coronary ostia were reimplanted to the neo-aortic root using slit incisions in the vessel walls or by utilizing the trap-door technique. The neo-pulmonary root was reconstructed using a single, pantaloon-shaped, untreated, autologous pericardium patch. When present, ventricular septal defects were closed using a synthetic patch. Primary aortic coarctation was repaired by resection and direct end-to-end anastomosis. A hypoplastic aortic arch, if present, was incised longitudinally, a direct anastomosis between the back wall of the aortic arch and the descending was performed, and the front wall was enlarged using an untreated autologous pericardial patch or a decellularized xenograft patch.

## 3. Results

During the study period, 127 consecutive arterial switch operations were performed at our center. A summary of our patients’ demographic and operative characteristics is provided in Table 1.

The majority (79.5%) of patients underwent neonatal arterial switch. At the time of operation, the median age was 13 days (IQR, 9–22), and the median weight was 3.5 kg (IQR, 3.2–3.9). The most frequent prior procedure was a Rashkind balloon atrial septostomy performed in 62 (48.8%) patients. Three patients (2.4%) underwent a staged repair. One (0.8%) underwent arterial switch due to obstruction in the systemic venous return after Senning repair. The most frequent concomitant cardiovascular malformations in our cohort were aortic coarctation (7.9%) and hypoplastic aortic arch (5.5%). One patient operated on for a Taussig-Bing transposition had a concomitant interrupted aortic arch, which was repaired during the arterial switch operation. Ninety-three (73.2%) patients had normal coronary patterns (1LCx-2R), according to the modified Leiden convention [18]. In total, 16 unique coronary patterns were observed among 35 (27.6%) patients with anomalous coronary patterns (Table 2). Four (11.4%) of these patients had an intramural coronary artery.

### 3.1. Surgical Mortality

During the entire study period, the surgical mortality rate in our center was 23.6% (Table 1). The high surgical mortality rate can be attributable to the poor results at the beginning of the congenital heart program. The first attempts to surgically repair transposition of the great arteries in Vilnius University Hospital Santaros Clinics Cardiothoracic Surgery Center were made in 1977. The first successful arterial switch operation in our center was performed in 1994 [19]. 

These unsatisfactory results drove an initiative to increase patient safety and improve surgical outcomes of patients treated for transposition of the great arteries and other complex heart malformations in Vilnius University Hospital Santaros Clinics Cardiothoracic Surgery Center. This led to the establishment of an international knowledge transfer program led by Professor Marcus Haw from the Wessex Cardiothoracic Center in Southampton, which took place between 1999 and 2002.

To analyze the significance of the knowledge transfer program to the surgical mortality rates, our arterial switch operation experience was divided into three surgical periods. The first “pre-program” period took place between 1977 and 1998, during which 17 patients underwent arterial switch operations for transposition of the great arteries. During the second “knowledge transfer program” period, between 1999 and 2002, 24 patients underwent the arterial switch operation. During the third “post-program” period, from 2003 to present, 86 patients have undergone the arterial switch operation. During these three surgical periods, surgical mortality decreased from 88.24% in the first period to 41.7% in the second period and to 5.81% in the third period (Fisher’s exact test *p*-value < 0.001). When compared to the first surgical period, the surgical mortality rate decreased significantly in the second and third periods (Table 3).

The sustainability of the program is shown by the fact that the surgical mortality rate keeps improving, even after the program had ended. Surgical mortality in the third period is lower when compared to the surgical mortality rate of the second period (Fisher exact test *p*-value < 0.001). During the last 10 years, 49 patients underwent arterial switch operations with a surgical mortality rate of 4%. Thirty-three patients were operated on for transposition of the great arteries and intact ventricular septum, and only one (3%) patient died. None of the eight patients operated on for transposition and ventricular septal defect died. One (12.5%) out of eight patients operated on for a Taussig-Bing anomaly died.

### 3.2. Early Morbidity

Two (1.6%) patients underwent left hemidiaphragm plication after the arterial switch operation due to left diaphragm paresis on post-switch day 12 and 22. One patient (0.8%) underwent bidirectional Glenn shunt due to an inadequately sized right ventricle on post-switch day five. These three patients were operated on due to failure to extubate. After an uneventful additional repair, all three patients were successfully extubated and discharged. At the time of this article, all three patients were in good health. None of the surviving patients in our cohort required mechanical circulatory support or heart transplant in the early period after the arterial switch operation.

### 3.3. Survival Analysis, Late Mortality, and Morbidity

A total of 97 (76.4%) patients survived the arterial switch operation in our center. The median follow-up time of all surviving patients in our study was 10.1 years (IQR, 5.69–14.53). A summary of our cohort characteristics during the late post-operative period is provided in Table 4.

Overall, six (7.7%) patients died during the late follow-up period. One patient had a choking accident that required cardiopulmonary resuscitation. During the event, this patient experienced a brain injury, which led to chronic respiratory failure and death in the follow-up period. Three patients died of infectious complications: one due to massive bilateral pneumonia, another due to infectious endocarditis, and the third from a pulmonary embolus, which formed in the right leg due to septic knee and hip joint arthritis. The thrombus was dislodged during an angiographic exam. Two patients died in an outside hospital due to unknown causes. Thirteen (13.4%) surviving patients had their follow-up visits more than two years ago. These patients either grew up or migrated to a different country. In both cases, they were managed by their local cardiologists. To the best of our knowledge, these patients were alive and well.

**Figure 1:**
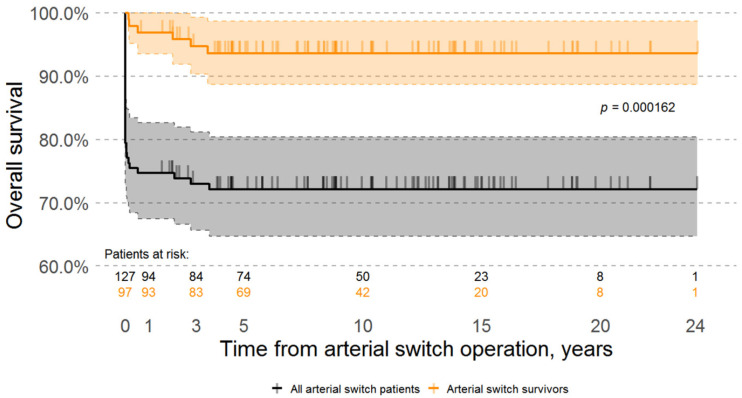
Kaplan-Meier survival curves. Time 0 is the day of arterial switch operation. Black curve represents survival of all pa-tients in our cohort and includes the early surgical mortality. Orange curve represents the survival curve of hospital survivors (excluding those patients who died during the 30 days post arterial switch operation). Y-axis scale was al-tered to range from 60% to 100%.

The overall estimated survivals in our cohort (including early in-hospital mortality) were 72.44%, 71.65%, 71.65%, 71.65%, and 71.65% at 3, 5, 10, 15, and 20 years respectively; while the overall survival estimates of the surviving patients were 96.9%, 94.9%, 93.8%, 93.8%, 93.8%, 93.8% at 1, 3, 5, 10, 15, and 20 years (Figure 1).

**Figure 2:**
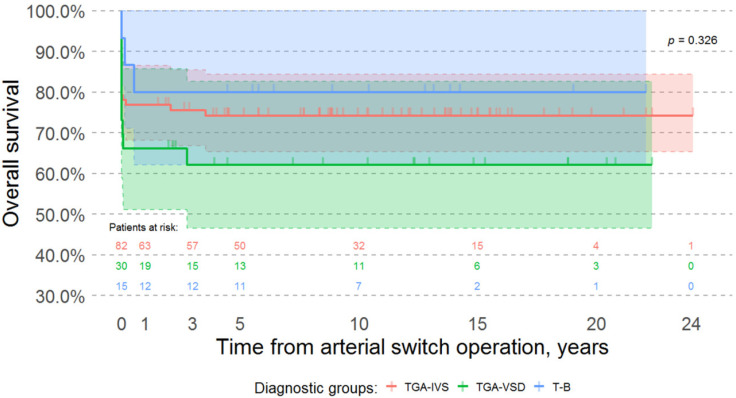
Kaplan-Meier survival curves of different diagnostic groups (including early surgical mortality). Time 0 is the day of arterial switch operation. There is no significant difference in overall survival between different diagnostic groups (Log-rank test *p*-value is 0.326). Y-axis scale was altered to range from 30% to 100%. Abbreviations: TGA-IVS, D-transposition of the great arteries with intact ventricular septum; TGA-VSD, D-transposition of the great arteries with ventricular septal defect; T-B, Taussig-Bing anomaly.

There was no significant difference in the overall survival of patients with different forms of transposition of the great arteries (Log-rank test *p*-value = 0.326) (Figure 2).

**Figure 3:**
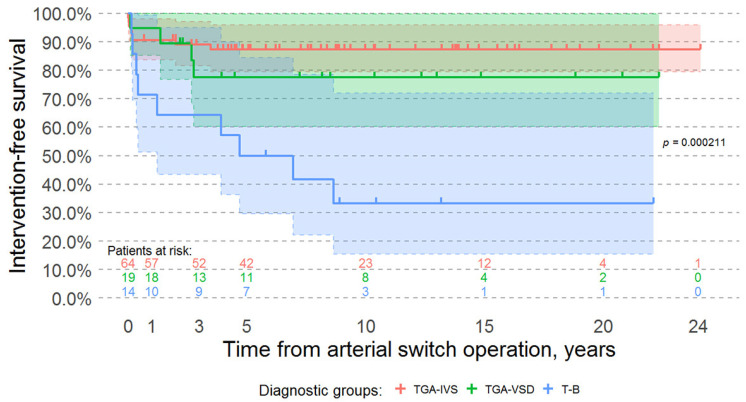
Kaplan-Meier intervention-free survival curves of surviving patients. Time 0 is the day of the arterial switch operation. In our cohort, intervention-free survival is significantly worse among patients with a Taussig-Bing anomaly when compared to patients with transposition of the great arteries and intact ventricular septum and patients with transposition of the great arteries and a ventricular septal defect (Log-rank test *p*-value = 0.000211). Abbreviations: TGA-IVS, D-transposition of the great arteries with intact ventricular septum; TGA-VSD, D-transposition of the great arteries with ventricular septal defect; T-B, Taussig-Bing anomaly.

However, intervention-free survival was significantly better for patients with transposition of the great arteries and both intact ventricular septum and ventricular septal defects when compared to patients with a Taussig-Bing anomaly (Log-rank test *p*-value < 0.001) (Figure 3).

Risk factors for surgical mortality after arterial switch operation in our cohort included longer aortic cross-clamp time and being operated on before the knowledge transfer program (crude odds ratio, 1.0076 and 28.5, respectively; adjusted odds ratio, 1.02 and 41.06, respectively). Both factors were significant predictors of surgical mortality in our cohort (multivariate analysis Wald’s test *p*-values = 0.026 and < 0.001, respectively) (Table 5).

Risk factors for late death or need for reintervention include a complex form of transposition of the arteries, concomitant repair of aortic arch obstruction during arterial switch operation, coronary pattern other than 1LCx-2R, longer aortic cross-clamp time, and longer length of stay (crude odds ratio, 5.38, 13.8, 2.06, 1.02, and 1.06, respectively; adjusted odds ratio, 3.05, 10.64, 0.43, 1.003, and 1.03, respectively). However, only concomitant repair of aortic arch obstruction during arterial switch operation was a significant risk factor on multivariate logistic analysis (Wald’s test *p*-value = 0.032) (Table 6).

### 3.4. Late Neurological Outcomes

During the last follow-up visit, 88 (96.7%) of the 91 currently alive patients had no neurological issues. Neither patients nor their parents/guardians had any complaints of anxiety, depression, or abnormal behavior. All patients attended kindergarten or school. Their neurological development and learning capabilities did not differ from those of the healthy Lithuanian population. Three of the surviving patients developed brain injuries and neurological development issues. However, only one case may be directly attributed to the arterial switch operation. One patient had an intracranial hemorrhage during birth and underwent an arterial switch with prior neurological damage. One patient underwent a staged repair and had brain damage after a pulmonary banding procedure. The third patient underwent a staged repair and had no signs of neurological issues prior to the arterial switch operation; however, after the operation, this patient showed signs of neurological deficit, and radiologic studies confirmed the presence of a brain injury.

### 3.5. Late Cardiovascular Outcomes

The most common cardiovascular issues after arterial switch operation in our cohort were neo-aortic valve regurgitation and root enlargement. On discharge, only nine patients had neo-aortic regurgitation. During follow-up, neo-aortic regurgitation was present in 55 patients. Moderate and greater neo-aortic regurgitation was present in eight patients. All patients with neo-aortic regurgitation had preserved left ventricle function, were asymptomatic, and at the time of writing this article, did not require any treatment. Neo-aortic annulus and neo-aortic root enlargement (Z-scores > +2) were present in 36 and 45 patients, respectively. However, there were no associations between neo-aortic regurgitation and neo-aortic annulus or root enlargement.

Eleven patients required re-intervention due to right ventricle outflow tract obstruction. Nine of these patients were treated for supravalvular neo-pulmonary stenosis, while two patients underwent bi-directional Glenn shunt due to inadequate size of the right ventricle. The majority of surviving patients (68 patients) had a peak Doppler gradient < 36 mmHg in the neo-pulmonary artery. Only five patients had peak Doppler gradients > 64 mmHg in the neo-pulmonary artery. Two patients developed new-onset aortic coarctation after discharge following an arterial switch. Five patients who underwent concomitant aortic arch obstruction repair during arterial switch required additional treatment during follow-up due to repeat aortic arch obstruction. After arterial switch operations, 16 patients required 41 additional treatment procedures (Table 7).

During routine follow-up visits, no surviving patients had any coronary circulation-related issues. No patients developed heart failure, required mechanical circulatory support or heart transplantation. Six patients had hemodynamically insignificant residual ventricular septal defects and pulmonary and systemic blood flow ratios between 1.1–1.3. One patient developed pulmonary hypertension one month after an uneventful neonatal arterial switch. At the time of this article, the patient was stable at home with a specific three-drug therapy.

## 4. Discussion

Arterial switch operation is considered a state-of-the-art gold standard for the treatment of various forms of transposition of the great arteries. Surgical mortality of this procedure, when performed in a large high-expertise center, is usually reported in single digits [5,6,7,8,9,10,11]. In some centers, the surgical mortality rate is nearly 0% [13]. As with every surgical procedure, a learning curve must be passed. This is also true with the arterial switch procedure. In their article, Hutter et al. stated that at the beginning of the arterial switch program in their center, mortality rates were as high as 60%, which were gradually reduced to as low as 4%, with an overall mortality rate of 15% in 195 patients operated on between 1977 and 2000 [12]. Similarly, Khairy et al. also reported an initial mortality rate of 15.1%, which gradually decreased to 3.9% in 400 patients operated on between 1983 and 1999. Other centers have reported similar experiences [10]. Michalak et al., in their article, reported an overall surgical mortality of 7.4% in a cohort of 715 patients operated on between 1991 and 2015, and a 2.9% surgical mortality rate among the patients operated on between 2001 and 2015 [7]. There are some centers that have reported extremely low mortality rates throughout [9]. The majority of publications report data from large centers in which hundreds of arterial switch operations were performed. However, similar surgical mortality rates can be achieved in small and medium case-load centers. Shim et al. reported an astonishing mortality rate of 2.2% among a cohort of 139 patients who underwent an arterial switch operation between 1995 and 2014 in South Korea [15]. However, other authors from small and medium case-load centers have reported overall surgical mortalities ranging from 4.8 to 28% [14,20,21,22]. Similar to the experience of large centers, surgical mortality rates tend to decrease with time [14,15,20,21]. Shim et al. reported that two out of three patients who died during the 30 post-operative days were operated on at the beginning of the arterial switch program [15]. Puras et al. reported a decrease in surgical mortality rates from 19% in patients operated on between 1985 and 1998 to 8% in patients operated on between 1998 and 2010 [21]. Manso et al. documented a drastic decrease in surgical mortality from 44% to 25% to 16% in patients operated on between 1995 and 2004, 2005–2008, and 2009–2014, respectively [14]. The gradual improvement of survival may be attributed to the learning curve of the congenital cardiac team. In our experience, the learning curve was extended and required external intervention. There are several major reasons why this occurred. The first attempts to treat patients with the arterial switch operation were made when Lithuania was still under strict Soviet rule. The congenital heart team had limited resources and practically no possibilities to observe and consult surgeons and teams in western countries. Limited economic resources and medical equipment led to a significant number of delayed diagnoses. At the time these patients were referred for surgical treatment, their conditions were severe. After the collapse of the Soviet Union, when Lithuania regained independence, the major issue became the lack of finance of the newly developing health system of a new country. This is reflected in the extremely high surgical mortality rates seen until 1998.

In our cohort, only longer aortic cross-clamp time and repair, prior to 1999, were significant risk factors for surgical mortality (crude odds ratios were 1.0076 and 28.5, respectively, and adjusted odds ratios were 1.02 and 41.06, respectively). Transposition of the great arteries with ventricular septal defects and a Taussig-Bing anomaly (complex form of transposition of the great arteries), anomalous coronary patterns, patient age and weight, and concomitant aortic arch anomalies were not risk factors in our cohort. The main reason is that the majority of patients with complex transposition, anomalous coronary arteries, and concomitant arch anomalies were operated on with the help of our foreign colleagues during the knowledge transfer program or independently by our team after the program. Blume et al. identified that prior pulmonary artery banding without a Blalock-Taussig-Thomas shunt, delayed sternal closure, side-by-side position of the great arteries, aortic arch repair prior arterial switch operation, prematurity, Taussig-Bing transposition, right ventricle hypoplasia, lower weight, longer circulatory arrest, and longer total cardiopulmonary bypass time were independent factors for early mortality [23]. However, in their experience, only prior aortic arch repair, lower birth weight, right ventricle hypoplasia, longer circulatory arrest, and longer total cardiopulmonary bypass were significant factors on multivariable analysis. Other groups found similar risk factors for surgical mortality, including concomitant resection of left ventricle outflow tract obstruction, presence of aortic arch obstruction, need for extracorporeal membrane oxygenation, surgical experience, prior procedures, and post-operative re-exploration due to hemorrhage [5,6,7,9,10,12,14].

Late mortality of arterial switch survivors is almost universally exceptionally low, despite the size and expertise of the center [7,9,10,12,14,15,21,24,25,26,27]. This is also true in our experience. Overall, excellent results in large centers, and increasing good results in small and medium centers, lead to an increasing population of arterial switch survivors. The majority of arterial switch survivors are in good cardiac status (left ventricle ejection fraction is normal, New York Heart Association class 1 or 2), and usually only individual patients have less than good cardiac status [5,6,9,10,12,21,22,24,28,29]. However, this population of patients requires long-term follow-up, as up to 25% may develop late post-switch complications that require additional treatment [5,7,9,10,11,12,14,20,25,26,30,31]. The most common late arterial switch-related complications are neo-aortic root enlargement, neo-aortic valve regurgitation, stenosis of the main neo-pulmonary artery or its branches, and repeat aortic arch obstruction. The incidence of neo-aortic root enlargement and neo-aortic valve regurgitation is similar in high volume and small-to-medium volume centers. Both neo-aortic root enlargement and neo-aortic valve regurgitation seldom require any treatment [11,14,15,21,22,24,32]; however, patients who required treatment for neo-aortic regurgitation were older [11,32]. This may be seen in our experience. Few patients had neo-aortic regurgitation on discharge. During the follow-up period, 55% of our cohort had neo-aortic regurgitation, and only eight patients had moderate or above neo-aortic regurgitation. Currently, no patients in our cohort required additional treatment for neo-aortic root enlargement or neo-aortic regurgitation. Neo-pulmonary stenosis is the most common cause of additional treatment for right-sided lesions in patients after arterial switch operation. Usually, patients with neo-pulmonary stenosis require more than one additional catheter-based interventional or surgical treatment procedure [6,7,15,21,31,33,34].

In the early days of arterial switch procedures, early post-operative coronary-related complications were attributed to the poor surgical transfer of coronary ostia. Currently, the technique of coronary ostia transfer is so evolved that the coronary artery pattern has minimal influence on the success of the arterial switch operation [23]. However, as the nature of the operation involves the excision and translocation of coronary ostia, arterial switch survivors may be prone to coronary artery-related complications during the early and late post-operative period [12,30]. Lim et al., in their article, reported three patients who required reoperation due to coronary obstruction [26]. Angeli et al. reported that reoperations for coronary lesions were the main cause of additional treatment after arterial switch operation [34]. Jung et al. documented five patients who underwent seven reoperations due to coronary lesions [22]. As the patients who develop coronary lesions after arterial switch operations may be asymptomatic, and the impact of atherosclerosis on the development of coronary lesions for these patients is unclear, these patients require close follow-up [30]. Some surgical centers may choose to perform a routine angiographic evaluation of the coronary arteries during follow-up, as described by Shim et al. [15]. In our experience to date, none of the surviving patients have had any symptoms or clinical or echocardiographic data suggesting coronary lesions. Currently, we employ watchful waiting follow-up tactics and do not perform routine coronary angiography.

As the survival of arterial switch operation improves and the number of surviving patients increases, the impact of the operation on the neurological development and quality of life of these patients becomes more important. Andropoulos et al., in their prospective observational study of 30 arterial switch patients, found that 10 patients exhibited pre-operative neurological injury visible on MRI, and 13 patients displayed evidence of new neurological injury on a post-operative MRI. Despite the fact that some of their patients had below-average neurodevelopment scores, the neurodevelopment scores were within normal of the whole cohort [35]. The authors have also identified potential risk factors for lower neurodevelopment scores, which include lower pre-operative and intra-operative cerebral oximetry values and larger cumulative doses of sedatives and opioids. In their experience, balloon atrial septostomy, the presence of a new brain injury on post-operative MRI, and the total dose of volatile anesthetics were not associated with neurodevelopment outcomes. However, the authors suggest that the low patient number may have influenced these results [35]. The prevalence of psychiatric and neurological conditions, such as attention deficit hyperactivity disorder, depression, anxiety, and disruptive behavior among teenagers and young adults who underwent arterial switch operation as neonates, are higher compared to healthy populations [36,37]. In our experience, only three patients had a neurological impairment, and only one of the cases may be directly attributed to the arterial switch operation. All other surviving patients had similar neurological development compared to the general Lithuanian population. None of our teenager or adult patients complained of depression or anxiety. None of the parents of younger patients had any complaints regarding the behavior of their children.

The overall outcomes of the arterial switch operation greatly depend on the cumulative experience of the congenital heart surgery center and the surgeon performing the procedure [38,39]. The prevalence of congenital heart disease depends on the size of the population and the birth rate in that population. As in our case, there might be a single congenital heart center in a small population with a low birth rate. In these conditions, acquiring the necessary expertise can be difficult. There are three options available to deal with these clinical situations: the patient may be transferred to a high expertise center, a team from a high expertise center may be brought in, or the patient may be operated on by a local team trained in an international knowledge program. Some authors suggest that it may be justified to concentrate patients in high expertise and volume centers [40]. Patient concentrations at high expertise and volume centers, however, may not always be feasible. Patient transfer to a high expertise high-volume center may be impossible due to high costs, political instability, or global health crises. The same difficulties may impede outside team travel to the patient. Training a local team may be a long process, however—as the old Chinese proverb says, “Give a man a fish and you will feed him for a day; teach a man how to fish and you will feed him for a lifetime.”—it is the only option beneficial to the whole population. In a 2015 interview, Professor Sir Magdi Yacoub said “The arterial switch operation is state of the art operation for the transposition of the great arteries, but the problem is that this is available to less than 10% of population of the world. This is unacceptable. There are ways and means of dealing with this problem which must be implemented in the near future. We cannot ignore this massive divide between those who have and those who have not”. Yacoub et al., in their 2017 article, mention that the number of congenital heart surgeons and specialized centers in developing countries remain critically low. The authors state that international cooperation may help to improve outcomes, shorten learning curves, and enhance the sustainability of the congenital heart surgery program in a given population [41]; this is true in our experience. The international knowledge transfer program between our center and our British colleagues was described in detail by Baliulis et al. [42]. This program helped to improve short- and long-term outcomes of the arterial switch operation in our center. The outcome improvement remains sustainable years after the program. Professor Richard Jonas advocates such knowledge transfer programs. In his article, he denotes that a long-term education and support model may be more beneficial and can provide medical help to more patients than a medical tourism sponsorship for single patients [43]. In order for the knowledge transfer program to be successful, there are pre-existing conditions that need to be met, such as existing infrastructure for performing complex cardiac procedures, presence of adequately trained staff, motivation of the local staff to adapt to new standards of practice, enthusiasm for the program by all participating members, adequate financial support for the program, continuous evaluation of results and adjustment, adequate program duration ensuring stable performance of the trainees, selection of complex congenital cardiac cases for surgery by the combined team, and maintenance of close relationship between the teams in order to discuss the management of the most complex cases [42,43].

## 5. Study Limitations

This study is a retrospective analysis of a single centers’ experience and involves a small number of patients over a long time period. As this study is of a retrospective nature, it poses all limitations characteristic to retrospective studies.

## 6. Conclusions

The surgical treatment of transposition of the great arteries by means of an arterial switch with good results can be possible in low-to-medium volume congenital heart surgery centers. International knowledge transfer programs between high-expertise, high-volume congenital heart centers, and low-to-medium volume congenital heart centers may help to shorten the learning curve and improve early and late outcomes after the arterial switch. The risk factors for surgical mortality and intervention-free survival in low-volume surgical centers are similar to those in high-volume centers. Late arterial switch-related complications are similar among different-sized congenital heart centers.

## Figures and Tables

**Table 1:** Demographic and operative characteristics of patients at time of operation.

Variable	TGA-IVS	TGA-VSD	T-B	Total
*n* (%)	82 (64.6)	30 (23.6)	15 (11.8)	127 (100)
Female, *n* (%): Male, *n* (%)	37 (45.1): 45 (54.9)	14 (46.7): 16 (53.3)	8 (53.3): 7 (46.7)	59 (46.5): 68 (53.5)
Median age, days (IQR)	11 (8–15)	26 (12–75)	21 (10–61)	13 (9–22)
Age ≤ 28 days, *n* (%)	77 (93.9)	16 (53.3)	8 (53.3)	101 (79.5)
Median weight, kg (IQR)	3.4 (3.1–3.8)	3.8 (3.3–4.4)	3.7 (3.5–4.2)	3.5 (3.2–3.9)
Coronary pattern *				
Normal, *n* (%)	66 (80.5)	24 (80.0)	2 (13.3)	93 (73.2)
Anomalous, *n* (%)	16 (19.5)	6 (20.0)	13 (86.7)	35 (27.6)
Intramural course ^#^, *n* (%)	3 (18.8)	1 (16.7)	0 (0.0)	4 (11.4)
Concomitant cardiovascular malformations				
AoCo, *n* (%)	2 (2.4)	0 (0.0)	8 (53.3)	10 (7.9)
HAA, *n* (%)	1 (1.2)	0 (0.0)	6 (40)	7 (5.5)
IAA, *n* (%)	0 (0.0)	0 (0.0)	1 (6.7)	1 (0.8)
Prior procedures				
BAS, *n* (%)	48 (58.5)	14 (46.6)	0 (0.0)	62 (48.8)
PAB, *n* (%)	0 (0.0)	1 (3.33)	2 (13.3)	3 (2.4)
Senning repair, *n* (%)	1 (1.22)	0 (0.0)	0 (0.0)	1 (0.8)
Surgical data				
Median CPB, min (IQR)	198 (174–227)	234 (213–262)	302 (260–354)	220 (181–245)
Median AoX, min (IQR)	125 (112–140)	133 (124–151)	174 (150–245)	130 (118–150)
Surgical mortality, %	22	36.7	6.7	23.6

* The coronary pattern is described in accordance to the modified Leiden convention [18]. ^#^ The percentages of the intramural coronary vessels are calculated from the number of anomalous coronary patterns in each group. Abbreviations: TGA-IVS, D-transposition of the great arteries with intact ventricular septum; TGA-VSD, D-transposition of the great arteries with ventricular septal defect; T-B, Taussig-Bing anomaly; *n*, count; IQR, interquartile range; AoCo, aortic coarctation; HAA, hypoplastic aortic arch; IAA, interrupted aortic arch; BAS, balloon atrial septostomy; PAB, pulmonary artery banding; CPB, cardiopulmonary bypass time; AoX, aortic cross-clamp time.

**Table 2:** Anomalous coronary patterns among arterial switch patients.

Coronary Pattern ^#^	TGA-IVS (*n* = 82) *n* (%)	TGA-VSD (*n* = 30) *n* (%)	T-B (*n* = 15) *n* (%)	Total (*n* = 127) *n* (%)
1LCx *–2R	1 (6.25%)	0 (0.0%)	0 (0.0%)	1 (2.86%)
1IB–2LCx, R	1 (6.25%)	0 (0.0%)	0 (0.0%)	1 (2.86%)
1L–2RCx	7 (43.75%)	2 (33.32%)	3 (23.1%)	12 (34.29%)
2R, LCx *	1 (6.25%)	0 (0.0%)	0 (0.0%)	1 (2.86%)
1LR–2Cx	1 (6.25%)	0 (0.0%)	1 (7.7%)	2 (5.71%)
1LCX, IB–2R	1 (6.25%)	0 (0.0%)	0 (0.0%)	1 (2.86%)
2LCx *, R	1 (6.25%)	0 (0.0%)	0 (0.0%)	1 (2.86%)
2L, RCx	1 (6.25%)	0 (0.0%)	0 (0.0%)	1 (2.86%)
1LCxR	1 (6.25%)	1 (16.67%)	1 (7.7%)	3 (8.57%)
1L, R–2Cx	1 (6.25%)	0 (0.0%)	0 (0.0%)	1 (2.86%)
2LCxR	0 (0.0%)	1 (16.67%)	0 (0.0%)	1 (2.86%)
1LCx–2R, R	0 (0.0%)	1 (16.67%)	0 (0.0%)	1 (2.86%)
1LCx *, R	0 (0.0%)	1 (16.67%)	0 (0.0%)	1 (2.86%)
1R–2LCx	0 (0.0%)	0 (0.0%)	5 (38.4%)	5 (14.29%)
1RCx, IB–2L	0 (0.0%)	0 (0.0%)	1 (7.7%)	1 (2.86%)
1IB–2LCxR	0 (0.0%)	0 (0.0%)	2 (15.4%)	2 (5.71%)

^#^ The coronary pattern is described in accordance to the modified Leiden convention [18]. Comma (,) indicates separate coronary orifices; asterisk (*) indicates intramural course of the vessel. Abbreviations: TGA-IVS, D-transposition of the great arteries with intact ventricular septum; TGA-VSD, D-transposition of the great arteries with ventricular septal defect; T-B, Taussig-Bing anomaly; *n*, count; 1, first coronary sinus; 2, second coronary sinus; L, left descending artery; Cx, circumflex artery; R, right coronary artery; IB, infundibular branch.

**Table 3:** Surgical mortality in different time periods.

	Surgical Mortality (%)	Fisher Exact *p*-Value against First Period
First period (1977–1998) (*n* = 17)	88.24	-
Second period (1999–2002) (*n* = 24)	41.7	0.0035
Third period (2003–2019) (*n* = 86)	5.81	<0.001

Abbreviations: *n*, count.

**Table 4:** Follow-up characteristics of surviving patients.

	TGA-IVS (*n* = 64)	TGA-VSD (*n* = 30)	T-B (*n* = 14)	Total (*n* = 97)
Late mortality (%)	4.7	5.3	14.3	6 (6.2)
FU visit > 2 years ago, *n* (%)	11 (17.2)	2 (10.5)	0 (0.0)	13 (13.4)
FU time, years (IQR)	9.13 (5.79–14.53)	11.24 (4.24–15.73)	11.5 (6.16–13.85)	10.1 (5.69–14.53)
Height, cm (IQR)	135 (106–160)	129 (105–161)	146 (116–158)	135 (106–160)
Weight, kg (IQR)	29 (17–50)	27.5 (16.8–55.3)	36.5 (18.4–51.5)	29 (16.8–50)
Cardiac function				
LV EF, % (IQR)	64 (62–66)	65 (62–70)	63 (61–66)	64 (62–68)
LV EF ≥ 55%, *n* (%)	49 (98.0)	15 (93.75)	12 (100)	76 (97.4)
LV EF 45–54%, *n* (%)	1 (2.0)	1 (6.25)	0 (0.0)	2 (2.6)
LVEDD, cm (IQR)	3.9 (3.4–4.6)	4.2 (3.2–4.6)	4.5 (3.8–4.8)	4 (3.4–4.6)
iLVEDD, cm/m^2^ (IQR)	3.8 (3.1–5.1)	3.8 (3.1–4.9)	3.8 (3.5–4.4)	3.8 (3.1–5.0)
Residual VSD, *n* (%)	-	3 (15.79)	3 (21.4)	6 (7.7)
NAA, cm (IQR)	1.9 (1.6–2.2)	1.83 (1.45–2.15)	2.1 (1.8–2.43)	1.9 (1.5–2.2)
NAA Z-score (IQR)	1.7 (0.9–3.1)	1.8 (1.42–2.3)	2.3 (1.3–4.1)	1.8 (1.1–3.1)
NAA Z-score ≥ 2, *n* (%)	23 (46)	6 (37.5)	7 (50)	36 (46.2)
NAS, cm (IQR)	2.6 (2.2–2.9)	2.6 (2.3–3.1)	3 (2.6–3.2)	2.6 (2.3–3.1)
NAS Z-score (IQR)	2 (1.1–2.8)	2.2 (1.7–2.7)	3.1 (2.2–3.4)	2.2 (1.3–2.9)
NAS Z-score ≥ 2, *n* (%)	26 (52)	9 (56.3)	10 (71.4)	45 (57.7)
NASTJ, cm (IQR)	2 (1.7–2.3)	1.8 (1.5–2.6)	2.1 (1.7–2.5)	2 (1.7–2.3)
NASTJ Z-score (IQR)	1.2 (0.5–1.7)	1.1 (0.2–2.1)	1.1 (0.8–1.9)	1.2 (0.4–1.9)
NASTJ Z-score ≥ 2, *n* (%)	6 (12)	4 (25)	2 (14.3)	12 (15.4)
NAVR on discharge, *n* (%)	2 (3.1)	4 (21.1)	3 (21.4)	9 (9.3)
NAVR during follow–up				
Trivial, *n* (%)	20 (40)	7 (43.75)	2 (14.3)	29 (37.2)
Mild, *n* (%)	10 (20)	5 (31.25)	3 (21.4)	18 (23.1)
Moderate, *n* (%)	5 (10)	0 (0.0)	2 (14.3)	7 (9)
Severe, *n* (%)	1 (2)	0 (0.0)	0 (0.0)	1 (1.3)
NPAPDG, mmHg (IQR)	12.1 (7.8–19.3)	12.3 (9.9–23)	15.2 (13–44.6)	13 (8.3–23)
NPAMDG, mmHg (IQR)	6 (4.9–11.7)	6.8 (5.1–11.2)	7.7 (6.7–31.6)	6.6 (5.1–11.8)
NPAPDV, m/s (IQR)	1.7 (1.4–2.2)	1.8 (1.6–2.4)	1.9 (1.7–2.6)	1.8 (1.4–2.4)
NPAPDG < 36, *n* (%)	45 (90)	14 (87.5)	9 (64.3)	68 (87.2)
NPAPDG > 64, *n* (%)	1 (2)	1 (6.3)	3 (21.4)	5 (6.4)
NPAPDV > 3, *n* (%)	4 (8)	2 (12.5)	3 (21.4)	9 (11.5)
Additional treatment due to				
NAAO, *n* (%)	1 (1.6)	1 (5.3)	0 (0.0)	2 (2.1)
RAAO, *n* (%)	0 (0.0)	0 (0.0)	5 (35.7)	5 (5.2)
RVOTO, *n* (%)	3 (4.7)	3 (15.8)	5 (35.7)	11 (11.3)

Abbreviations: TGA-IVS, D-transposition of the great arteries with intact ventricular septum; TGA-VSD, D-transposition of the great arteries with ventricular septal defect; T-B, Taussig-Bing anomaly; *n*, count; FU, follow-up; LV, left ventricle; EF, ejection fraction; LVEDD, left ventricle end-diastolic volume; iLVEDD, indexed left ventricle end-diastolic volume; VSD, ventricular septal defect; NAA, neo-aortic annulus; NAS, neo-aortic sinuses; NASTJ, neo-aortic sinotubular junction; NAVR, neo-aortic valve regurgitation; NPAPDG, neo-pulmonary artery peak Doppler gradient; NPAMDG, neo-pulmonary artery mean Doppler gradient; NPAPDV, neo-pulmonary artery peak Doppler velocity; NAAO, new-onset aortic arch obstruction; RAAO, repeat aortic arch obstruction; RVOTO, right ventricle outflow tract obstruction.

**Table 5:** Logistic regression analysis of risk factors for surgical mortality after arterial switch operation.

Variables	Data		Univariable Analysis			Multivariable Analysis		
	Event = 0 * (*n* = 97)	Event = 1 ^#^ (*n* = 30)	OR	95% CI	*p*-Value	OR	95% CI	*p*-Value
Female, *n* (%)	47(48.5)	12 (40)	1.06	0.44–2.6	0.892			
Age, days; M (IQR)	11 (8–19)	15 (11.3–38.8)	0.999	0.995–1.004	0.76			
Weight, kg; M (IQR)	3.5 (3.2–3.9)	3.45(3.3–3.9)	0.86	0.55–1.34	0.509			
Complex TGA, *n* (%)	33 (34)	12 (40)	1.39	0.56–3.45	0.485			
AAO, *n* (%)	15 (15.5)	2 (6.7)	0.5	0.11–2.34	0.376			
Coronary pattern other than 1LCx-2R, *n* (%)	30 (30.9)	5 (16.7)	0.59	0.2–1.72	0.333			
AoX, min; M (IQR)	126 (117–149)	139 (130–151)	1.0076	0.998–1.02	0.119	1.02	1–1.05	0.026
Surgery before the international knowledge transfer program, *n* (%)	2 (2.1)	15 (50)	28.5	5.61–144.89	<0.001	41.06	5.98–282.11	<0.001

* Event = 0 marks the patients who survived the early surgical mortality period. ^#^ Event = 1 marks the patients who did not survive arterial switch operation. Abbreviations: *n*, count; M, median; IQR, interquartile range; TGA, transposition of the great arteries; AAO, aortic arch obstruction; 1LCx-2R, normal coronary pattern according to the Leiden convention [18]; OR, odds ratio; CI, confidence intervals. Significant factors in multivariable analysis are marked in bold.

**Table 6:** Logistic regression analysis of risk factors for late mortality and reoperations after arterial switch operation.

Variables	Data		Univariable Analysis			Multivariable Analysis		
	Event = 0 * (*n* = 76)	Event = 1 ^#^ (*n* = 21)	OR	95% CI	*p*-Value	OR	95% CI	*p*-Value
Female, *n* (%)	39 (51.3)	8 (38.1)	0.63	0.23–1.7	0.37			
Age, days; M (IQR)	11 (8–17.25)	13 (9–28)	0.999	0.994–1	0.76			
Weight, kg; M (IQR)	3.5 (3.2–3.9)	3.6 (3.3–4.0)	1.01	0.72–1.4	0.97			
Complex TGA, *n* (%)	20 (26.3)	13 (61.9)	5.38	1.87–15.5	0.002	3.05	0.71–13.16	0.135
AAO, *n* (%)	5 (6.6)	10 (47.6)	13.8	3.9–48.7	<0.001	**10.64**	**1.23–99.19**	**0.032**
Coronary pattern other than 1LCx-2R, *n* (%)	21 (27.6)	9 (42.9)	2.06	0.75–5.7	0.162	0.43	0.08–2.31	0.324
AoX, min; M (IQR)	125 (90–141)	140 (110–203)	1.02	1.01–1.04	0.002	1.003	0.98–1.03	0.785
Length of stay, days; M (IQR)	25 (20–31)	30 (22.75–39)	1.06	1.01–1.12	0.03	1.03	0.97–1.1	0.36

* Event = 0 marks the patients who did not undergo the compound endpoint (including death during the late peri-operative period or additional interventional or surgical treatment due to late arterial switch complications). ^#^ Event = 1 marks the patients who underwent the compound endpoint (including death during the late peri-operative period or additional interventional or surgical treatment due to late arterial switch complications). Abbreviations: *n*, count; M, median; IQR, interquartile range; TGA, transposition of the great arteries; AAO, aortic arch obstruction; 1LCx-2R, normal coronary pattern according to the Leiden convention [18]; OR, odds ratio; CI, confidence intervals. Significant factors in multivariable analysis are marked in bold.

**Table 7:** Additional treatment procedures during early and late post-operative periods.

Procedure	Number (%)	
Diaphragm plication	2	(5)
Right ventricle outflow tract obstruction repair	2	(5)
Bi-directional Glenn shunt	2	(5)
Neo-pulmonary artery balloon dilation	11	(27)
Neo-pulmonary artery stenting	1	(2)
Neo-pulmonary artery surgical repair	6	(15)
New aortic coarctation surgical repair	1	(2)
New aortic coarctation balloon dilation	1	(2)
Aortic re-coarctation balloon dilation	13	(32)
Aortic re-coarctation stenting	2	(5)
Total	41 in 16 patients	

## Data Availability

The data presented in this study are available on request from the corresponding author.

## Abbreviation

IQR	interquartile range
